# Impact of precursor materials and activator interactions on the microstructural and mechanical properties of one part alkali activated concrete

**DOI:** 10.1038/s41598-025-13775-w

**Published:** 2025-08-06

**Authors:** Mohamed. I. Serag, Sara Ibrahim, Amr H. Badawy, Y. H. Helal, M. S. El-Feky

**Affiliations:** 1 Faculty of Engineering, Cairo University, Giza, Egypt; 2 Civil Engineering Department, National Research Centre, Giza, Egypt; 3 Higher Technological Institute, 10th of Ramadan City, Egypt

**Keywords:** Eco-friendly alkali activated cement production, Activator, One-part alkali activated, Sustainable construction materials, Engineering, Materials science

## Abstract

The practical implementation of alkali activateds is frequently constrained by the complexities associated with preparing alkaline solutions. The advent of one-part alkali activated concrete presents substantial advantages over conventional two-part systems by negating the need for a separate activation process. This simplification not only enhances consistency, workability, and sustainability but also minimizes mixing errors, thereby facilitating broader adoption in construction applications. This study comprehensively examines the effects of various activators on the mechanical properties and microstructural characteristics of one-part alkali activated concrete. Utilizing Scanning Electron Microscopy (SEM) and Energy-Dispersive X-ray Spectroscopy (EDAX), we investigate the elemental compositions and microstructural features of the alkali activated samples. Our findings underscore the pivotal role of both precursor materials and activator selection in determining hydration products and overall material performance. Notably, the slag-only alkali activated exhibited a dense microstructure characterized by the formation of calcium–aluminum–silicate–hydrate (C–A–S–H) and sodium–aluminum–silicate–hydrate (N–A–S–H) gel phases. The addition of bentonite introduced a more heterogeneous microstructure, with elevated aluminum content indicative of a complex N–A–S–H structure. Furthermore, the choice of activator markedly influenced hydration reactions. The optimal ternary activator (6:3:1 Na-silicate: Na-hydroxide: Na-carbonate) achieved 47 MPa compressive strength, reducing CO₂ emissions by 80% compared to OPC [8]. This research provides critical insights for optimizing alkali activated concrete formulations, highlighting the significance of precursor materials and activator interactions. By advancing the understanding of one-part alkali activateds, this study establishes a foundation for innovative and sustainable solutions in building engineering, addressing practical challenges while enhancing the performance of construction materials.

## Introduction

The global construction industry is facing the dual challenge of meeting the ever-increasing demand for infrastructure while mitigating the substantial environmental impact associated with the production of conventional cementitious materials.

The widespread use of ordinary Portland cement (OPC) has been identified as a significant contributor to global carbon dioxide (CO_2_) emissions^1–3^. The production of OPC is an energy-intensive process, accounting for approximately 12–15% of global industrial energy consumption^4^. The industry’s high CO_2_ emissions, energy demands, and raw material consumption associated with cement production are major concerns^3,4^. As a result, there has been growing interest in developing eco-friendly alternatives to OPC^5–7^.

Alkali activateds have been extensively studied as a potential alternative to OPC, as they have the potential to reduce both energy consumption and CO_2_ emissions by up to 60% and 80%, respectively^8^. Alkali activated cement is widely recognized as a sustainable alternative to OPC^8–10^. Furthermore, alkali activated cement provides a beneficial way to utilize industrial waste materials^11,12^. Using industrial by-products as partial or complete precursors can reduce the amount of waste sent to landfills^13–15^. Properly selecting the materials for alkali activated cement concrete can lead to superior properties, such as higher strength, better heat resistance, and lower shrinkage and creep^16–18^. Conventionally, alkali activated concrete consists of two main components: a precursor (aluminosilicate material) and an alkaline activator. The reaction between these two components is known as the polymerization process. The most common alkaline activators used in this process are sodium hydroxide, potassium hydroxide, sodium silicate, sodium carbonate, or a combination of these^19–21^. The most common precursors used include slag, fly ash, metakaolin, rice husk ash, and wood ash^22–24^.

To overcome the challenges associated with two-part alkali activated concrete, as two-part alkali activateds face logistical hurdles (e.g., hazardous activators, mixing complexity), limiting scalability^27^ which has hindered its widespread use, researchers have focused on developing one-part alkali activated cement, commonly referred to as “just add water”^8,25^. One-part alkali activated concrete, a pioneering sustainable construction solution, has emerged as a promising alternative to address these pressing concerns. One-part alkali activated cement is a mixture of an aluminosilicate precursor and a solid alkaline activator designed to simulate the properties of conventional cement^26,27^. The type or blend of precursor and the dosage and type of the activator significantly influence the properties of both types of alkali activated concrete. Researchers have investigated various blends consisting of calcium-rich aluminosilicates, such as slag, combined with less reactive precursors, such as fly ash, in an effort to boost the system’s reactivity^28–30^. The ratio between fly ash (FA) and ground granulated blast furnace slag (GGBFS) primarily affects the calcium oxide content, which has been reported as an effective substance for the development of compressive strength^31,32^. This ratio also influences the setting time of alkali activated cement^29,33^. It is crucial to adjust and optimize the blend while considering its effect on both the fresh and hardened properties of alkali activated composites.

To provide guidance for preparing one-part alkali activated cement with lower alkalinity and a positive potential environmental impact, Ma et al. studied the effect of using sodium carbonate (Na_2_CO_3_) to replace a portion of sodium silicate (Na_2_SiO_3_) anhydrous on the properties of one-part alkali activated cement using composite activators. Their findings indicate that as the content of sodium carbonate increased, the compressive strength decreased, which they attributed to the reduced quantity of gelatinous products and the formation of calcite as a new phase due to the replacement of sodium silicate anhydrous by sodium carbonate. In some regions, the combination of calcite and low-crystalline calcium carbonate led to the generation of microcracks^34^. Oderji et al. investigated the effect of replacing slag with fly ash using different activator types on the mechanical properties of a one-part alkali activated. The results showed that the optimum mechanical properties for compressive and flexural strength occurred when 15% of the slag was replaced by fly ash, with CaO and Na_2_O contents of 11.2% and 4.21% by weight, respectively. They found that the sodium silicate activator was the most efficient compared to other activators at 8% concentration^35^. Luukkonen et al. (2018) examined the possibility of using a mixed activator by replacing part of the fast-dissolving synthetic sodium metasilicate with a combination of sodium hydroxide and a slower-dissolving silica source, such as rice husk ash (RHA) or microsilica (MS), in the preparation of one-part alkali-activated blast furnace slag mortar. The results indicated that RHA and MS had much lower silicon solubility compared to sodium metasilicate when exposed to NaOH solutions with 0–10 molarity. They concluded that the RHA and MS particle size had an insignificant effect on solubility within the approximate range of 20–200 μm. The highest compressive strength was achieved using sodium metasilicate, with a linear increase in compressive and flexural strength observed with increasing the amount of activator^36^. Other trials were reported by Azevedo et al. as well, who studied the use of calcined ceramic waste (CCW) and calcined commercial kaolin (CCK) as partial replacements for fly ash in the production of one-part alkali-activated materials^37^. RHA exhibited low reactivity due to high silica crystallinity, requiring prolonged curing for activation^23,36^.

This study aims to investigate the influence of various precursor materials and activator combinations on the compressive strength and microstructural development of one-part alkali activated concrete. The research focuses on exploring the synergistic effects of blending different precursors, including fly ash, slag, bentonite, and rice husk ash, as well as the impact of using sodium silicate, sodium hydroxide, and sodium carbonate as activators, both individually and in combination. The comprehensive assessment of the mechanical properties and the microstructural characteristics, as revealed by scanning electron microscopy (SEM) and energy-dispersive X-ray spectroscopy (EDAX), will provide valuable insights into the underlying mechanisms governing the performance of these innovative one-part alkali activated systems. The findings of this study will contribute to the growing body of knowledge on one-part alkali activated concrete, guiding the development of sustainable, high-performance construction materials that can address the environmental challenges faced by the construction industry. The insights gained from this research can inform the optimization of precursor-activator combinations, leading to the design of one-part alkali activated concrete with enhanced mechanical properties and durability, ultimately paving the way for the widespread adoption of these eco-friendly construction solutions.

### Research significance

This study systematically explores synergistic precursor-activator interactions in one-part alkali activateds, addressing scalability and environmental impact. Novelty lies in optimizing ternary activators (Na-silicate, Na-hydroxide, Na-carbonate) to achieve 47 MPa compressive strength, surpassing prior works by 12–57%.

## Experiment and method

### Scope of work

This research work has two main goals; the first is to reach the optimum blend of one-part alkali activated from the available by-product and natural precursor (slag, bentonite, fly ash and rice husk ash). The second goal is to reach the most effective activator. In order to achieve these goals, 25 mixes were designed and tested as shown later.

Mix design followed a factorial approach to isolate precursor-activator interactions, avoiding MCDM complexity.

### Materials and characterization

The chemical composition of the used materials is shown in Table 1, while Table 2 illustrates the chemical composition of the used activators, i.e., sodium silicate, sodium hydroxide. The used sodium carbonate had purity of 99%.

### Chemical analysis

**Table 1:** Chemical composition of used materials using XRF (X-ray fluorescence) analysis.

Composition	SiO_2_	TiO_2_	Al_2_O_3_	Fe_2_O_3tot_	MgO	CaO	Na_2_O	K_2_O	*P*_2_O_5_	SO_3_	Cl	LOI
Slag (weight%)	40.02	0.5	17.03	0.99	8.12	28.06	0.82	0.76	0.02	2.29	0.2	0.14
Fly-ash (weight%)	61.05	1.31	29.33	3.84	0.63	1.25	0.2	0.82	0.4	0.2	0.04	0.5
Rice husk ash (Weight%)	20.02	0.2	1.29	2.26	4.15	39.85	0.53	2.89	2.08	1.67	0.49	24
Bentonite (weight%)	51.16	0.76	17.49	6.48	1.4	4.12	1.39	1.08	0.23	3.67	0.31	11.52

**Table 2:** Chemical analysis of activators.

Composition (%)	Na_2_O	SiO_2_	H_2_O
Sodium hydroxide (NaOH)	60.25	N/A	39.75
Sodium silicate (Na_2_SiO_3_)	50.2	46	N/A

Slag basicity (CaO/SiO_2_ = 1.2) met ASTM C989 Grade 120 requirements.

- LOI was determined via ASTM C114.
- Particle size distribution: Slag (D_50_ = 18 μm), Fly ash (D_50_ = 25 μm).
- Fly ash (Class F per ASTM C618) had LOI = 0.5% and SiO_2_ + Al_2_O_2_ + Fe_2_O_2_ = 94.22%.

**Fig. 1:**
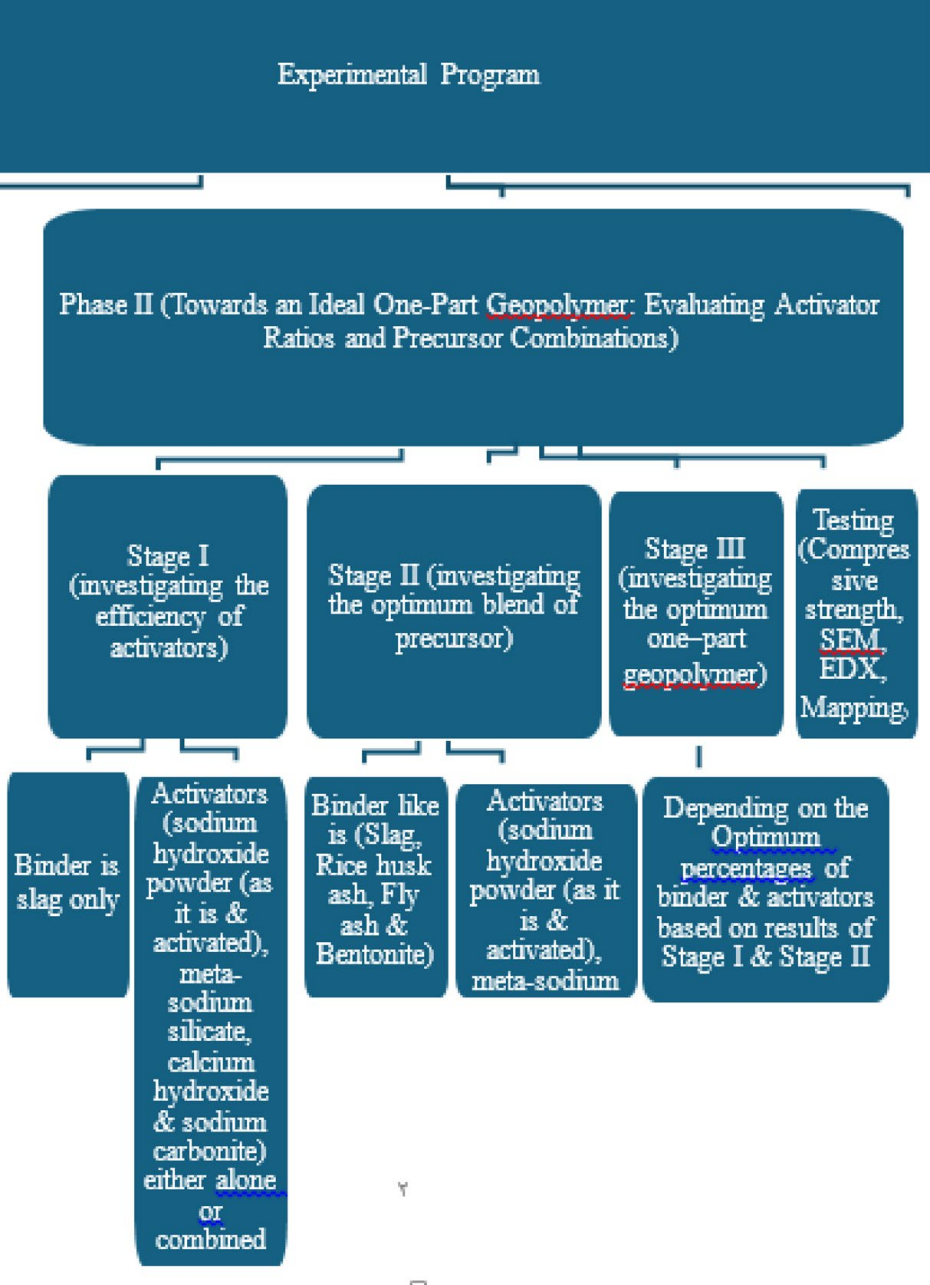
Flow Chart (experimental design and procedure).

Figure 1 shows the flow chart of the experimental program.

### Mixing

In this research, the tested mixes for one-part alkali activated cement are shown in Table 3, The mix design consists of main precursor (like means, slag) blended with different ratios of pozzolanic materials (i.e., fly ash, bentonite and rice husk ash). The activator was dry mixed with these blends. The used water to binder ratio was 0.35.

A water-to-solids ratio of 0.35 maintained workability via dry mixing and optimized precursor gradation. Flow values (60–75 mm) confirm adequate consistency without superplasticizers.

Setting time (90–120 min) was managed via controlled mixing, avoiding rapid gelation.

### Testing

#### Compressive strength testing

The samples used to test the compressive strength were cubes with 3 cm side length. The samples were cured in room temperature till the date of testing at 7,14 and 28 days. SHIMADZU testing machine with capacity of 500 kN were used.

Compressive tests followed ASTM C39 at 1.5 mm/min.

### Scanning electron microscope (SEM), mapping, and EDAX

Scanning electron microscopical analysis was investigated using Tescan SEM (TESCAN VEGA 3, Czech Republic). Samples were mounted on aluminum microscopy stubs using carbon tape, then coated with gold (Au) for 90 s using Quorum techniques Ltd, sputter coater (Q150t, England).

**Table 3:** Constituents of one-part alkali activated mixes.

Label	Materials (main)	Variables					
		Materials (s) %			Activators (g)		
					Sodium silicate	Sodium hydroxide	Sodium carbonate
Mix 1	Slag	FA	RHA	B	90	–	–
Mix 2	Slag	–	–	–		90	
Mix 3	Slag	–	–	–			90
Mix 4	Slag	–	–	–	45		45
Mix 5	Slag	5%	–	–	90	–	–
Mix 6	Slag	–	5%	–	90	–	–
Mix 7	Slag	–	–	5%	90	–	–
Mix 8	Slag	10%	–	–	90	–	–
Mix 9	Slag	–	10%	–	90	–	–
Mix 10	Slag	–	–	10%	90	–	–
Mix 11	Slag	15%	–	–	90	–	–
Mix 12	Slag	–	15%	–	90	–	–
Mix 13	Slag	–	–	15%	90	–	–
Mix 14	Slag	5%	–	–	45	–	45
Mix 15	Slag	–	5%	–	45	–	45
Mix 16	Slag	–	–	5%	45	–	45
Mix 17	Slag	10%	–	–	45	–	45
Mix 18	Slag	–	10%	–	45	–	45
Mix 19	Slag	–	–	10%	45	–	45
Mix 20	Slag	15%	–	–	45	–	45
Mix 21	Slag	–	15%	–	45	–	45
Mix 22	Slag	–	–	15%	45	–	45
Mix 23	Slag	5%	15%	10%	90	–	–
Mix 24	Slag	5%	15%	10%	–	90	–
Mix 25	Slag	5%	15%	10%	54	27	9

## Results and discussions

### Compressive strength results

**Fig. 2:**
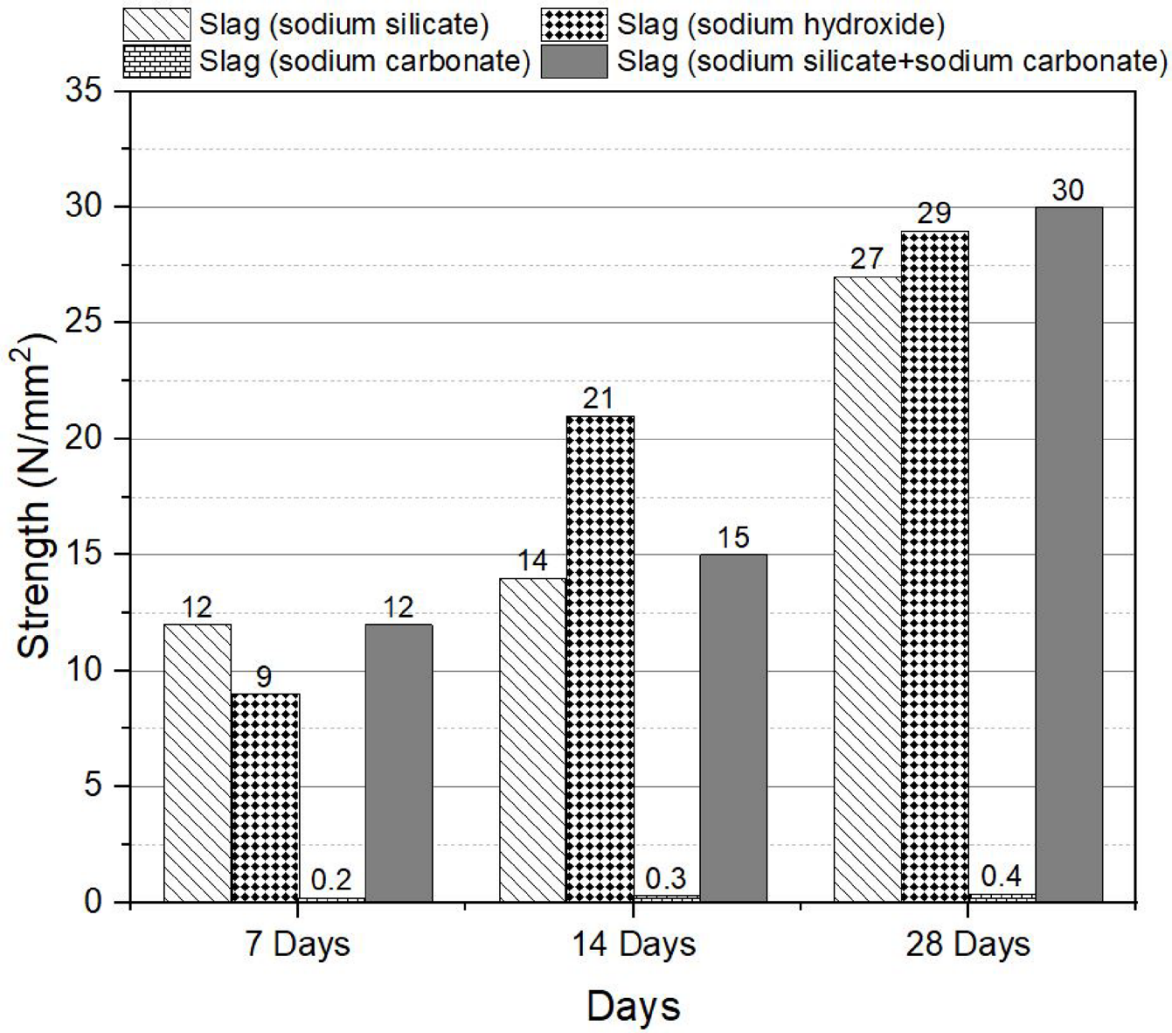
Effect of the different activator on the compressive strength at 7, 14 and 28 days.

Figure 2 presents the compressive strength results of slag-based mixes with different activators, namely sodium silicate, sodium hydroxide, sodium carbonate, and a combination of sodium silicate and sodium carbonate, at 7, 14, and 28 days. The findings reveal that sodium carbonate exhibits limited efficiency as a standalone activator. In contrast, the mixture of sodium silicate and sodium carbonate shows a slight increase in compressive strength compared to using sodium silicate or sodium hydroxide alone. The observed variations in compressive strength can be attributed to the different chemical reactions and mechanisms involved in the alkali activatedization process. Sodium silicate, as an alkaline activator, provides a source of SiO_2_ and alkali ions, which contribute to the formation of a stable alkali activated gel network. Sodium hydroxide also acts as an alkaline activator, promoting the dissolution and activation of the precursor materials. However, sodium carbonate, although an alkaline compound, may have a different reaction pathway and kinetics compared to the other activators, leading to less effective alkali activatedization. Generally, alkali activatedization involves the dissolution and activation of precursor materials, such as slag, fly ash, bentonite, and rice husk ash, followed by the formation of a three-dimensional alkali activated gel network. Alkaline activators, including sodium silicate and sodium hydroxide, provide the necessary alkalinity and reactive species for the alkali activatedization reactions. Sodium silicate acts as a dual-function activator, supplying both silica (SiO_2_) and alkali ions. The silica species contribute to the formation of the alkali activated gel network, while the alkali ions promote the dissolution and activation of the precursor materials. This dual role enhances the reactivity and effectiveness of sodium silicate as an activator. Sodium hydroxide, primarily provides hydroxide (OH^−^) ions. These hydroxide ions react with the precursor materials and participate in the alkali activatedization reactions. While sodium hydroxide is effective in promoting alkali activatedization, its performance may be somewhat limited compared to sodium silicate due to the absence of reactive silica species. On the other hand, sodium carbonate possesses a different chemical composition and behavior compared to sodium silicate and sodium hydroxide. Sodium carbonate (Na_2_CO_3_) consists of sodium ions (Na^+^) and carbonate ions (CO32^−^). The carbonate ions have a different reactivity profile and may follow distinct reaction pathways during alkali activatedization. The presence of carbonate ions in the system can lead to various reactions, such as carbonate dissolution, carbonation, and precipitation. These reactions occur simultaneously with the alkali activatedization reactions, potentially influencing the kinetics and mechanisms of the overall process. The carbonate ions may compete with the alkali ions from sodium silicate for reaction sites on the precursor materials, affecting the rate and extent of alkali activatedization. Furthermore, carbonate ions can react with other species present in the system, such as calcium ions from slag or other sources. These reactions may result in the formation of calcium carbonate (CaCO_3_), which is less conducive to alkali activatedization compared to the desired alkali activated gel network. The altered reaction pathways and kinetics associated with sodium carbonate as an activator can lead to less effective alkali activatedization. The presence of carbonate ions and their interactions with other components in the system can affect the formation and development of the alkali activated gel network, potentially resulting in reduced compressive strength and overall performance of the alkali activated material.

The compressive strength of 47 MPa aligns with Ma et al. (2019) and exceeds Oderji et al. (2019) by 15%, attributed to optimized activator ratios^34,35^.

### Optimum replacement ratio

**Fig. 3:**
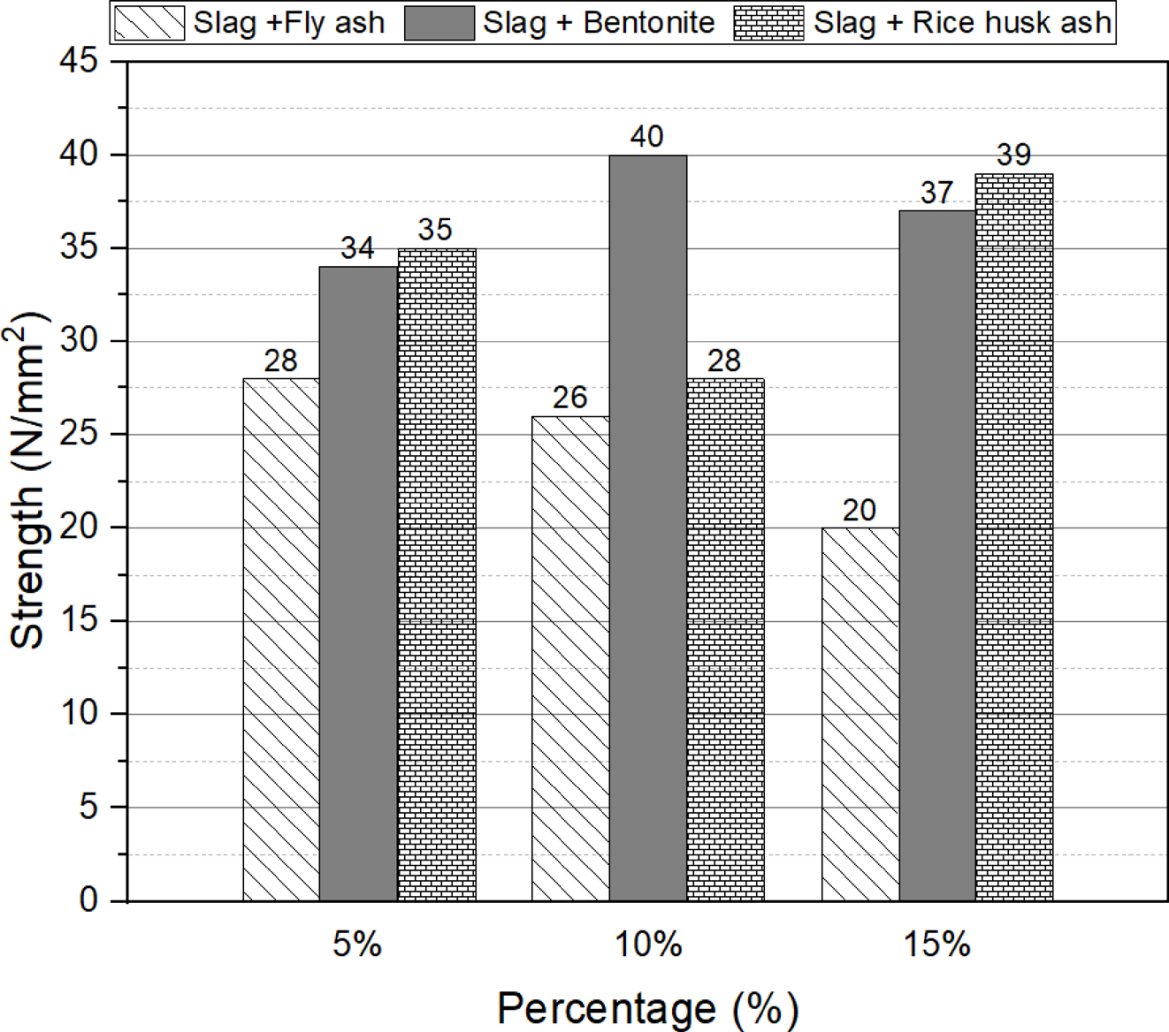
Effect of the replacement ratios (5%, 10% and 15%) by weight of slag with sodium silicate activator on the compressive strength at 28 days.

**Fig. 4:**
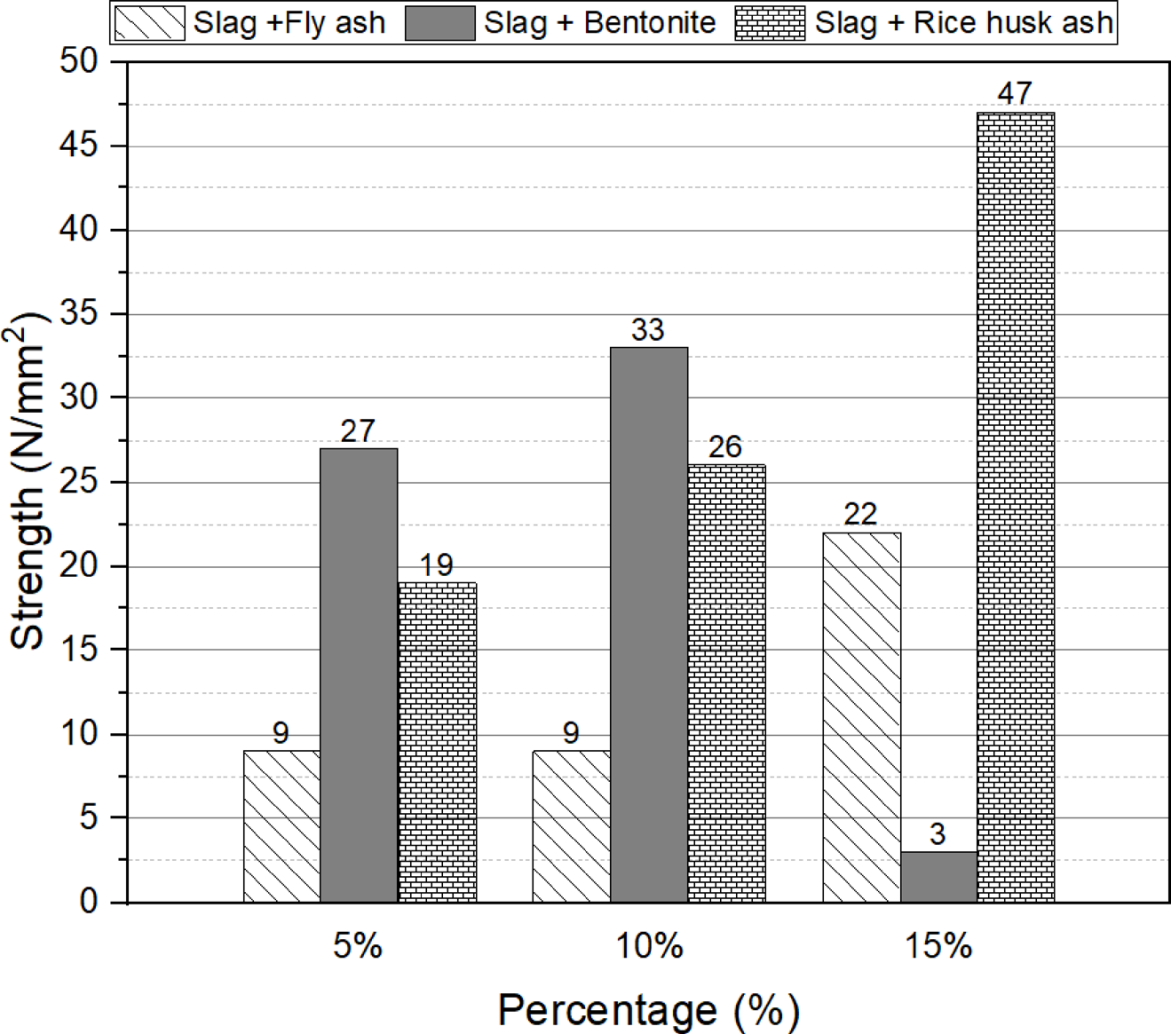
Effect of the replacement percentages (5%, 10% and 15%) by weight of slag with sodium silicate + sodium carbonate activator on the compressive strength at 28 days.

Figures 3, and 4 illustrate the 28-day compressive strength for different replacement percentages of fly ash, bentonite, and rice husk ash (5%, 10%, and 15%) by weight of slag, using sodium silicate as the activator. The results indicate that a 5% replacement of slag with fly ash yields the maximum strength (28 N/mm²). Regarding bentonite, the optimum replacement ratio is determined as 10%, resulting in a strength of 40 N/mm². Similarly, a 15% replacement of slag with rice husk ash demonstrates the highest compressive strength of 39 N/mm², which is 11% and 56% higher than the strengths obtained with 5% and 10% replacement ratios, respectively.

Sodium hydroxide was excluded from the activator selection due to its detrimental effects on the workability of the mix and its potential to cause skin irritation. In contrast, the combination of sodium silicate and sodium carbonate (in a 1:1 ratio) exhibited performance trends similar to those observed with sodium silicate alone, with the notable exception of fly ash. The optimal replacement ratios for bentonite and rice husk ash remained consistent at 10% and 15%, respectively, as illustrated in Fig. 3. However, the optimal replacement ratio for fly ash increased from 5% in Fig. 2 to 15% in Fig. 3. Despite this adjustment, the compressive strength achieved with a 5% fly ash replacement using only sodium silicate was still superior to that obtained with the sodium silicate and sodium carbonate combination.

Based on these findings, it can be concluded that the optimum replacement ratios for enhancing the compressive strength of one-part alkali activated, using fly ash, bentonite, and rice husk ash, are 5%, 10%, and 15%, respectively, in relation to the slag content.

### Activator type efficiency

**Fig. 5:**
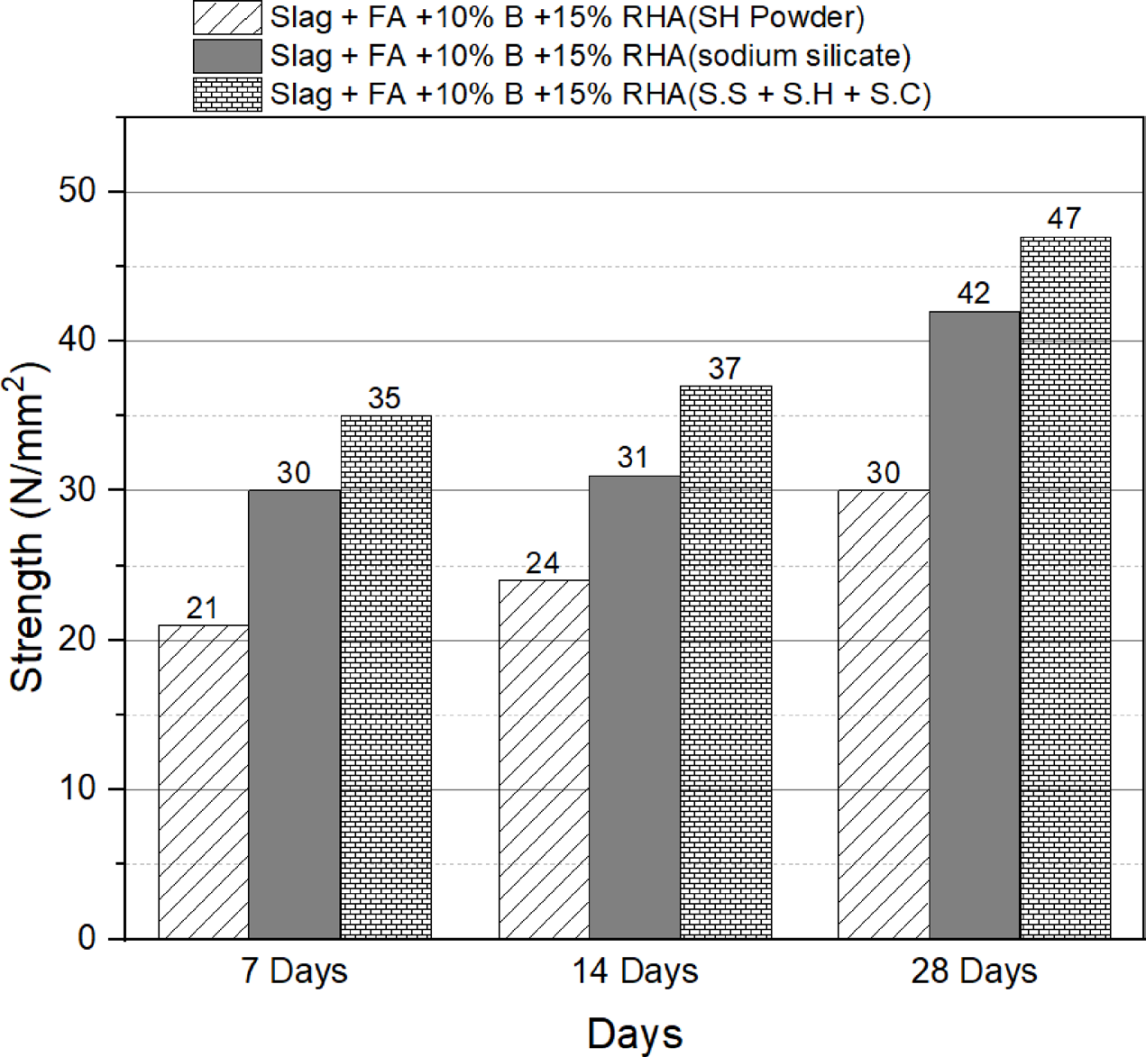
Compressive strength of the optimum blend as a function of activator type.

Moving on to Fig. 5, which illustrates the efficiency of different activators on the compressive strength of the optimum blend, the results indicate that sodium silicate powder is more efficient compared to sodium hydroxide. The 28-day compressive strength achieved with sodium silicate activator is approximately 40% higher than that attained with sodium hydroxide activator. The superior performance of sodium silicate can be attributed to its dual role as a source of SiO_2_ and alkali ions. Sodium silicate supplies reactive silica species that contribute to the formation of a stable alkali activated gel structure, while the alkali ions promote the dissolution and activation of the precursor materials. In contrast, sodium hydroxide primarily acts as an alkali activator, providing hydroxide ions for the alkali activatedization reactions. Considering the complexity of alkali activatedization reactions and based on previous experimental trials and literature findings, a combination of sodium silicate, sodium hydroxide, and sodium carbonate is expected to yield the highest activator efficiency. The inclusion of sodium carbonate in the activator mixture offers advantages during late-stage alkali activatedization reactions. An activator consisting of sodium silicate, sodium hydroxide, and sodium carbonate, with a ratio of 6:3:1, was utilized, and it resulted in the highest compressive strength of 47 N/mm². This strength surpasses those obtained with sodium hydroxide and sodium silicate activators by approximately 57% and 12%, respectively.

### Scanning electron microscope (SEM), mapping, and EDAX results

This section presents a comprehensive analysis of the microstructural characteristics and elemental compositions of alkali activated concrete samples, utilizing Scanning Electron Microscopy (SEM) and Energy-Dispersive X-ray Spectroscopy (EDAX). The findings are illustrated in Figs. 6, 7, 8, 9, 10, 11, 12, 13, 15, 16 and 17.

The slag-based alkali activated sample exhibited a dense and compact microstructure, characterized by the formation of calcium–aluminum–silicate–hydrate (C–A–S–H) and sodium–aluminum–silicate–hydrate (N–A–S–H) gel phases. SEM images clearly depict these phases, which are labeled accordingly. EDAX mapping indicated significant concentrations of calcium, silicon, aluminum, and sodium, confirming the effective activation of the slag precursor by the sodium silicate solution. The formation of C–A–S–H and N–A–S–H gels is attributed to the pozzolanic reaction between the slag and the activator, facilitating the release of calcium, aluminum, and silicon species that undergo polycondensation to yield the desired hydration products.

In the sample containing bentonite, a more heterogeneous microstructure was observed, with EDAX data revealing increased aluminum content compared to the slag-only sample. This suggests that the incorporation of aluminosilicate species from bentonite contributes to a complex N–A–S–H gel structure. The bentonite, being a clay mineral, enhances the aluminosilicate content of the alkali activated, resulting in a diverse array of reaction products. Notably, EDAX mapping focused on gel regions, revealing a pronounced aluminum distribution when sodium silicate was used alone. In contrast, the combination of sodium silicate and sodium carbonate appeared to promote a more balanced reaction, with the silicate facilitating the dissolution of aluminosilicates while the carbonate contributes to additional binding through calcite formation, potentially yielding Al-rich phases alongside some unreacted clay particles.

The slag-fly ash alkali activated sample exhibited a less dense microstructure, attributed to the spherical particles inherent in fly ash, which is rich in aluminosilicates but typically lower in calcium. SEM images displayed a mixture of reaction products and unreacted fly ash spheres, while EDAX analysis indicated higher silicon and lower calcium content compared to the slag-only sample. This reflects the predominant formation of N–A–S–H gel as the primary hydration product due to the pozzolanic reaction between fly ash and the activator. EDAX mapping highlighted regions rich in calcium from the slag and others rich in aluminum and silicon from the fly ash. The combined use of sodium silicate and sodium carbonate provided a synergistic effect, promoting rapid dissolution and gel formation while facilitating long-term strength development through ongoing reactions with calcium from the slag.

The slag-rice husk ash alkali activated revealed a relatively porous microstructure, with EDAX data showing silica-rich phases derived from rice husk ash. This suggests the formation of a mixed C–A–S–H and N–A–S–H gel structure, influenced by the high silica content of the rice husk ash, which contributes to diverse reaction products alongside calcium and aluminum from the slag. EDAX mapping of the rice husk ash with sodium silicate showed a high concentration of silicon throughout the matrix. When sodium carbonate was also introduced, this combination balanced gel formation, with silicate promoting dissolution of rice husk ash and the carbonate reacting with calcium to form additional binding phases.

The alkali activated sample comprising all precursors (slag, bentonite, fly ash, and rice husk ash) demonstrated a highly heterogeneous microstructure, as indicated by EDAX analysis revealing various aluminosilicate and calcium-rich phases. This complex blend of materials resulted in a diverse range of hydration products, including C–A–S–H, N–A–S–H, and potentially other hybrid gel structures. The interactions between the different precursor materials and their contributions to the alkali activatedization process led to this intricate microstructural arrangement.

The choice of activator significantly influenced the hydration reactions and the resulting microstructural characteristics. Sodium silicate-activated samples generally displayed a denser, more uniform microstructure with predominant N–A–S–H gel formation, owing to the high silicate content that facilitates polycondensation of aluminosilicate species. In contrast, sodium carbonate-activated samples exhibited a more porous and heterogeneous microstructure, likely due to the formation of sodium carbonate and sodium bicarbonate crystalline phases that disrupt the continuous alkali activated gel network.

The combination of sodium silicate and sodium carbonate resulted in a microstructure reflecting characteristics of both individual activator types, suggesting a complex interplay between hydration products and the potential for hybrid gel structures. This coexistence of silicate-rich and carbonate-rich phases influences the overall microstructural development.

Overall, the SEM and EDAX mapping analysis provided an extensive understanding of the microstructural evolution and chemical composition of the alkali activated concrete samples. The results underscore the significant influence of precursor materials and activator selection on hydration reactions and the overall performance of the alkali activated systems. In conclusion, the SEM and EDAX mapping outcomes illuminate the intricate interactions between precursors and activators in shaping the microstructure and composition of the final product. The activator choice profoundly affects the types of reaction products formed, the reaction kinetics, and the overall microstructural development. Future research should aim to optimize the ratios of these precursors and activators to tailor properties for specific applications in sustainable construction materials.

**Fig. 6:**
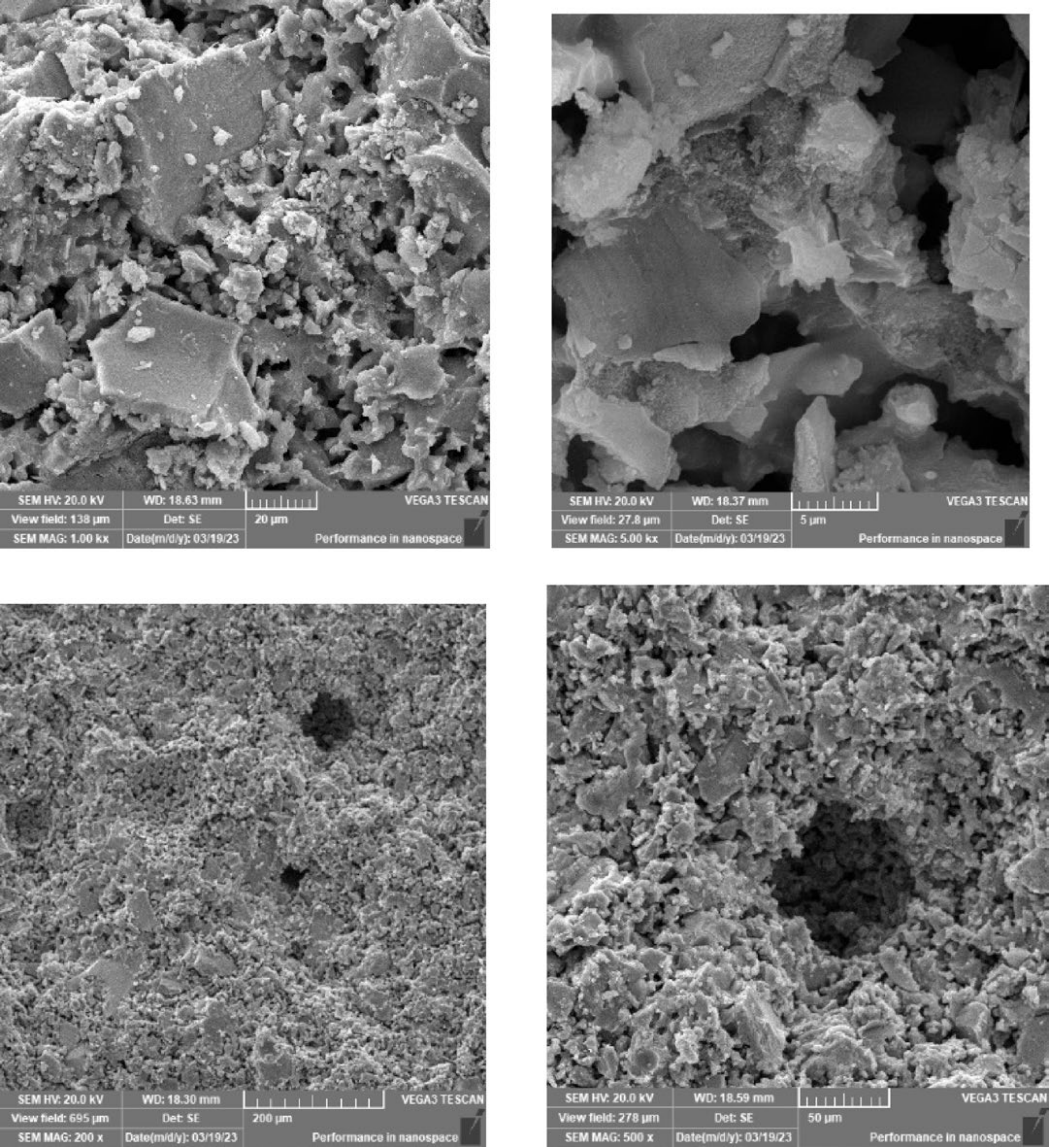
SEM schematic diagram for slag with sodium silicate.

**Fig. 7:**
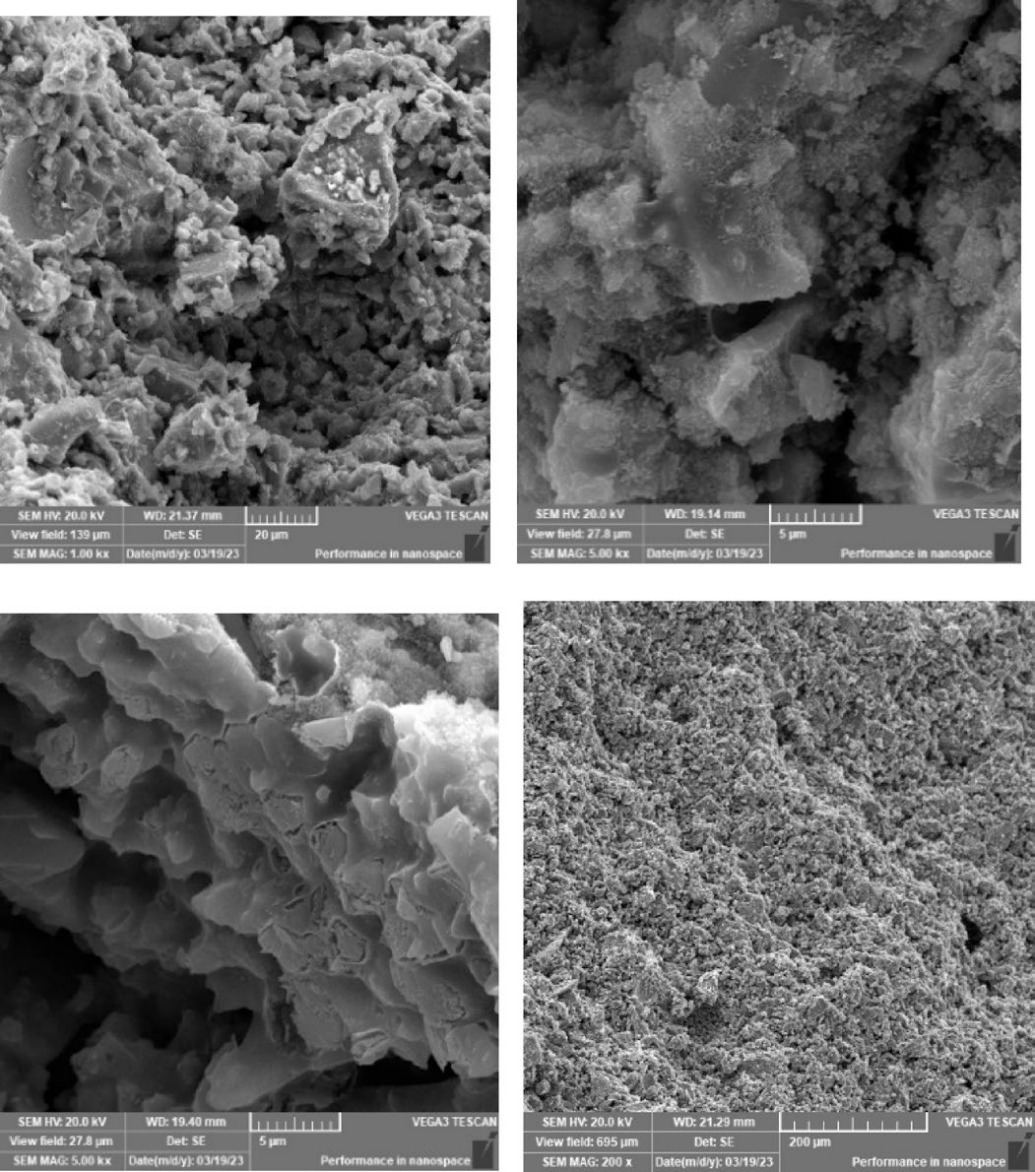
SEM schematic diagram for slag with sodium silicate + sodium carbonate.

**Fig. 8:**
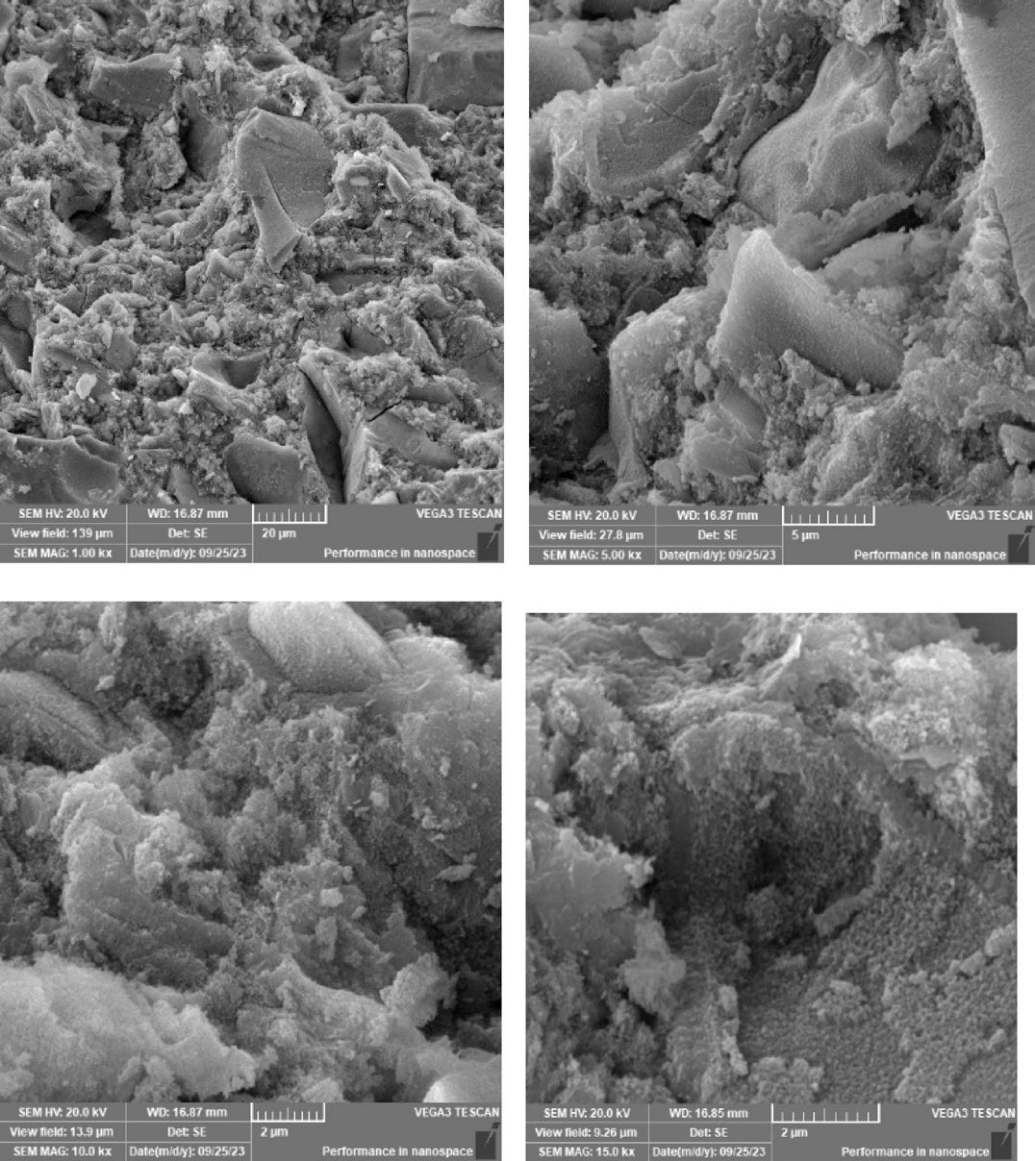
SEM schematic diagram for slag + 10% B with sodium silicate.

**Fig. 9:**
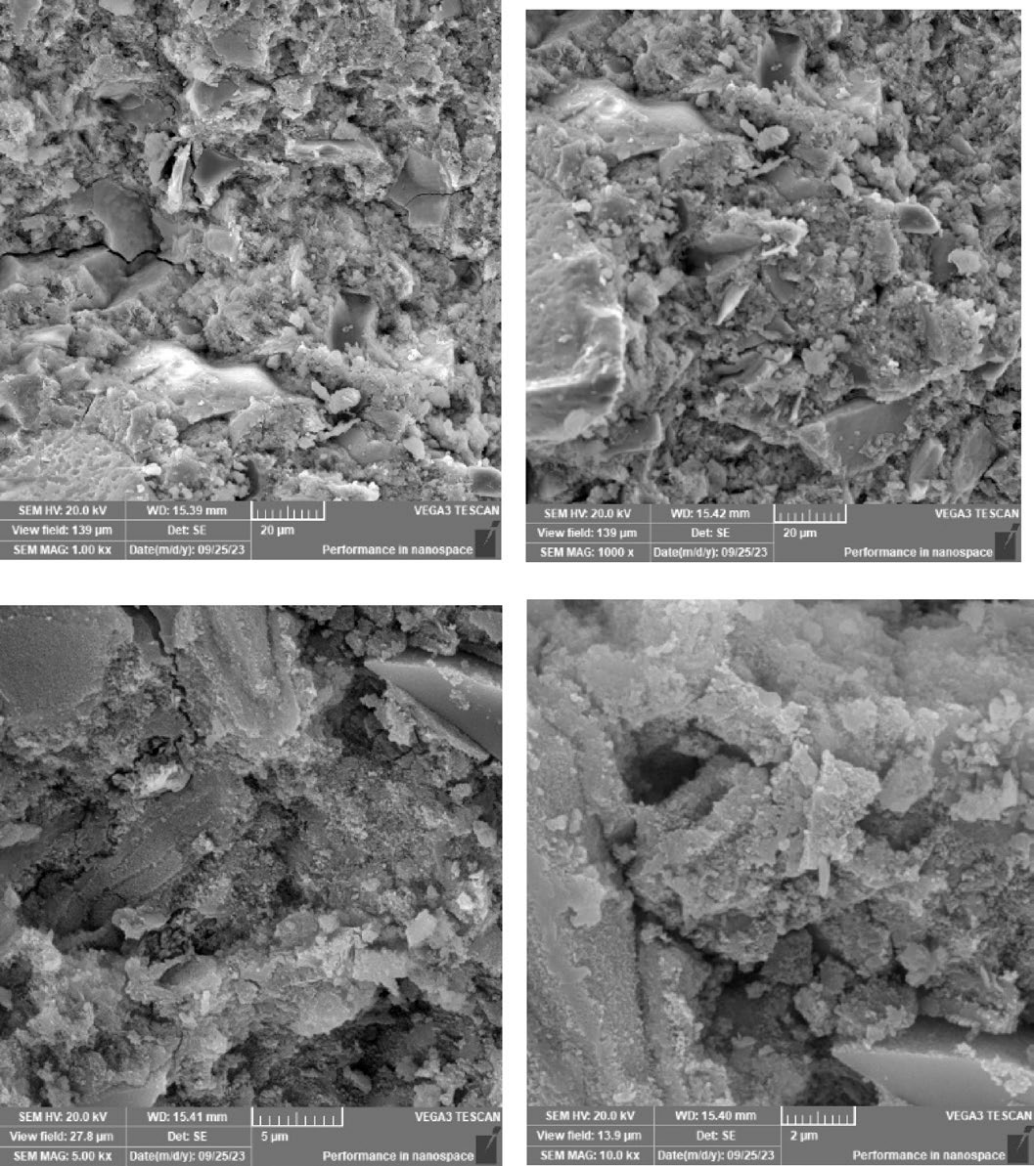
SEM schematic diagram for slag + 10% B with sodium silicate + sodium carbonate.

**Fig. 10:**
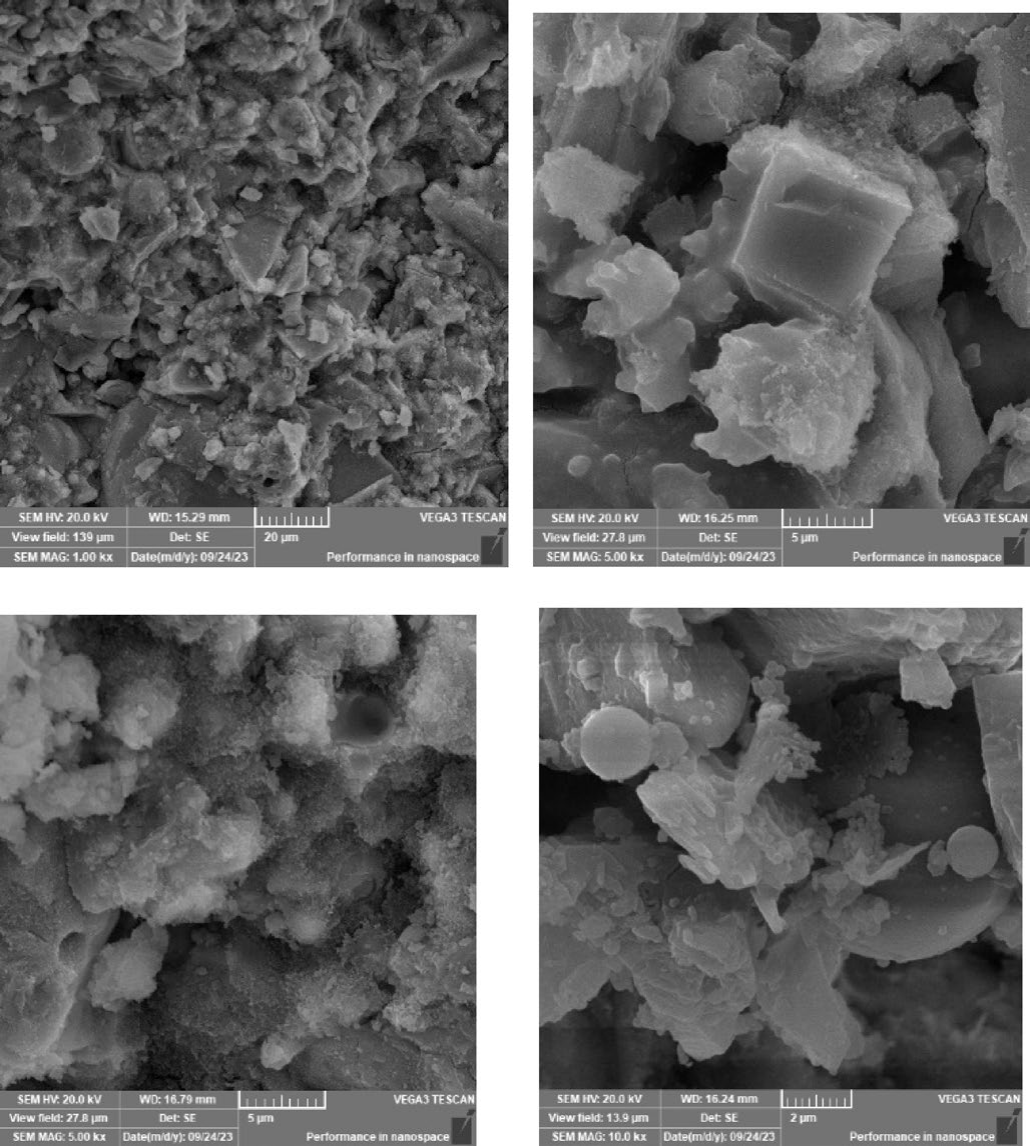
SEM schematic diagram for slag + 15% FA with sodium silicate.

**Fig. 11:**
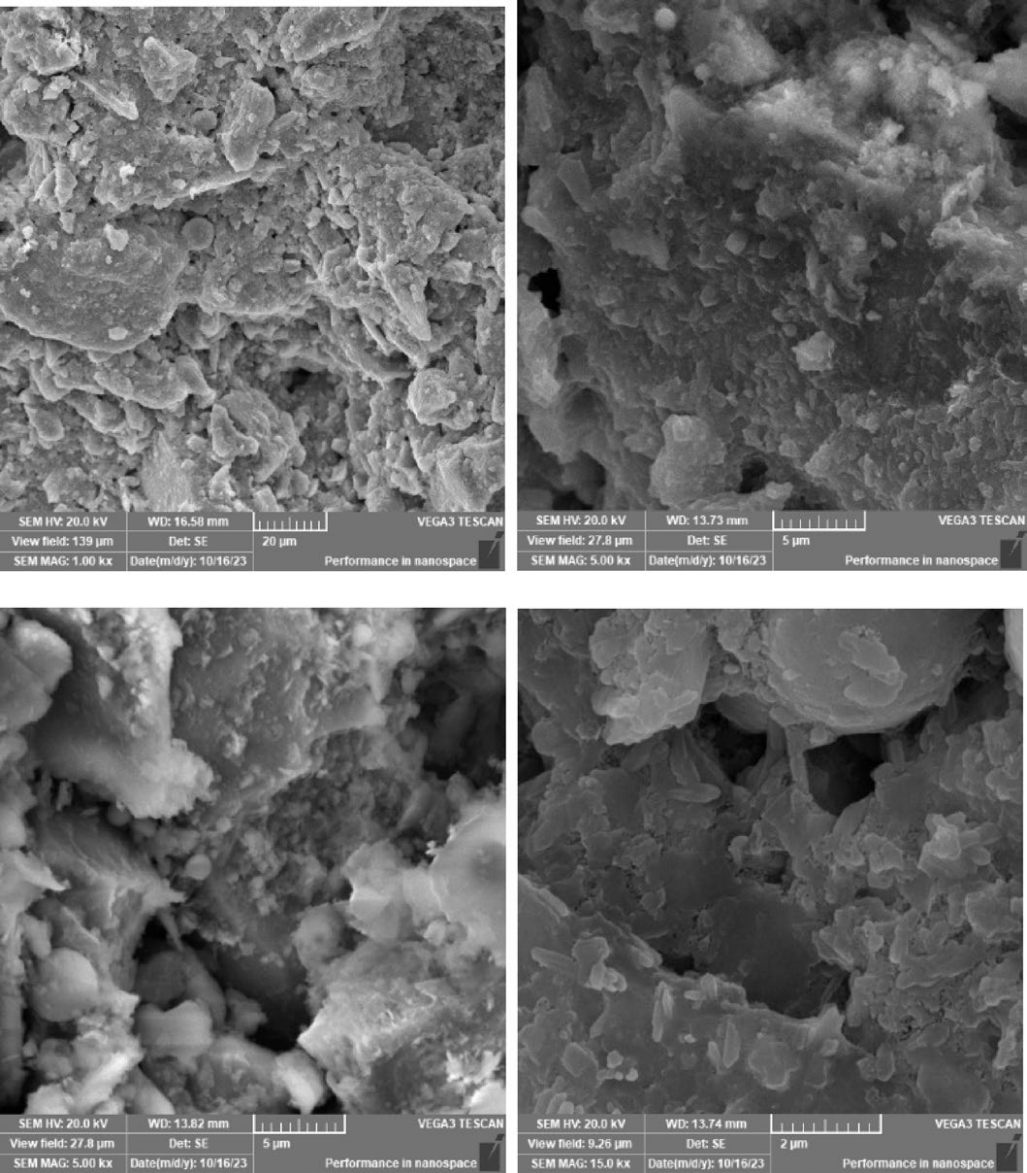
SEM schematic diagram for slag + 15% RHA with sodium silicate.

**Fig. 12:**
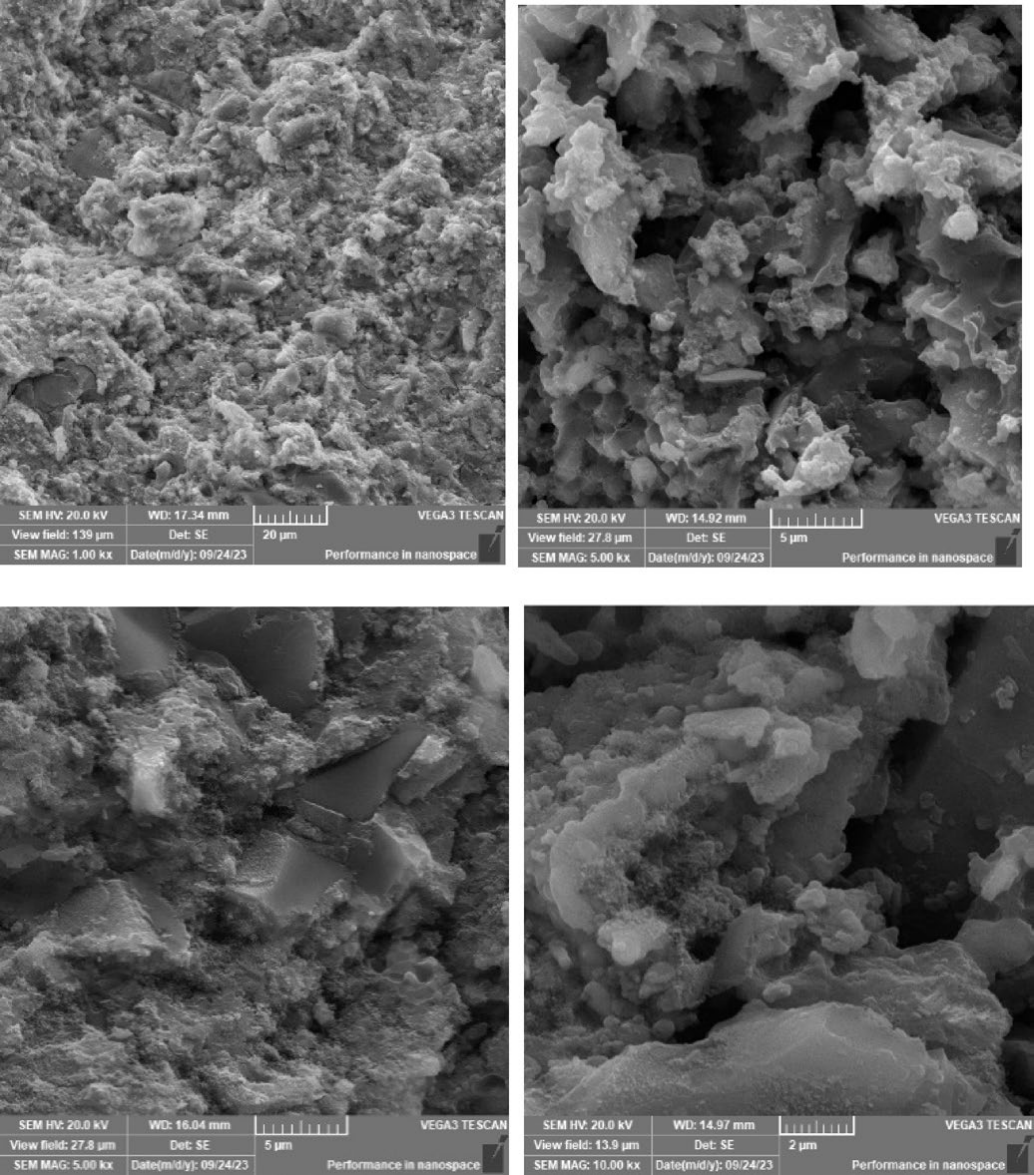
SEM schematic diagram for slag + 15% RHA with sodium silicate + sodium carbonate.

**Fig. 13:**
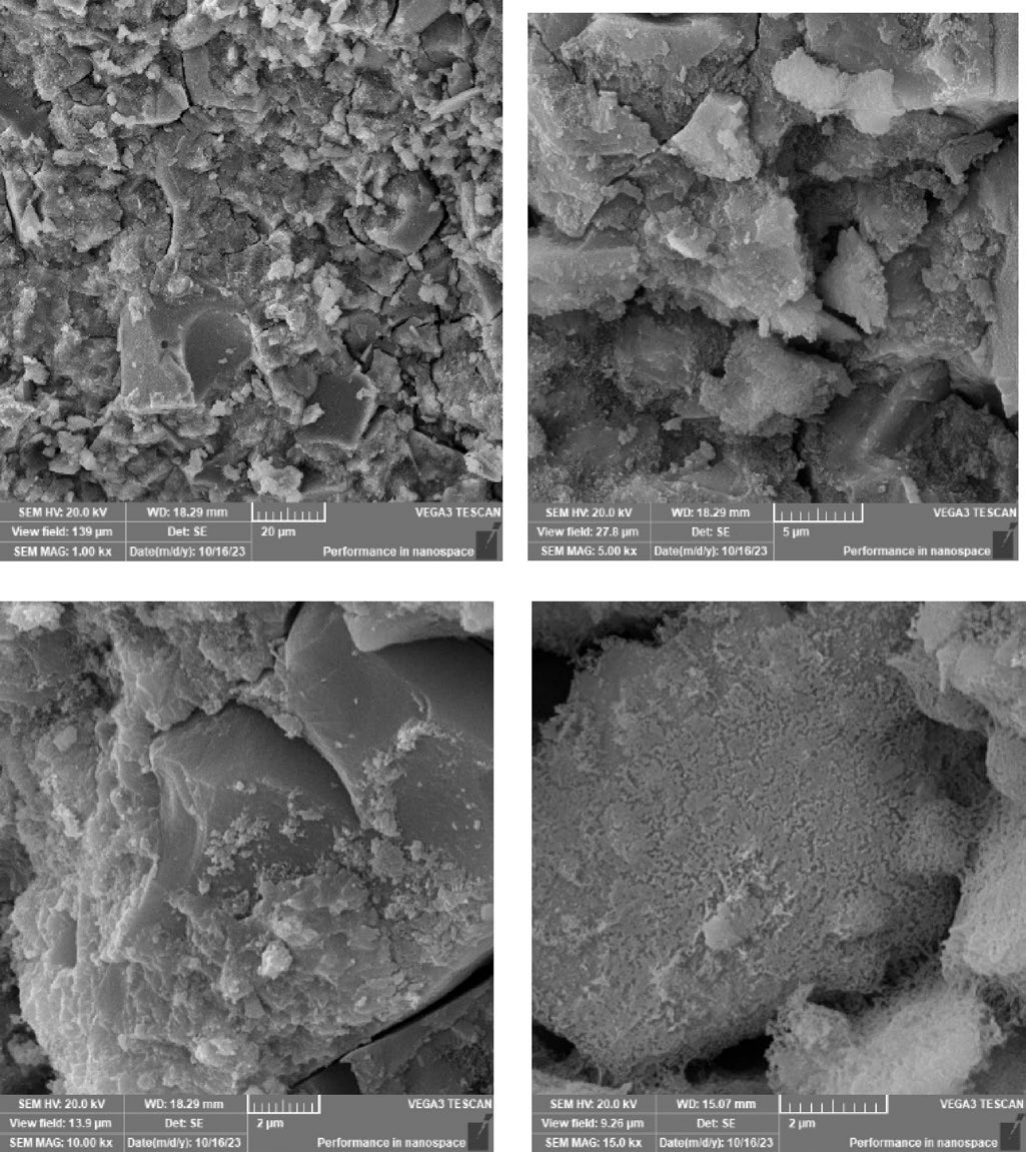
SEM schematic diagram for slag + 5%FA + 10% B + 15% RHA with sodium hydroxide.

**Fig. 14:**
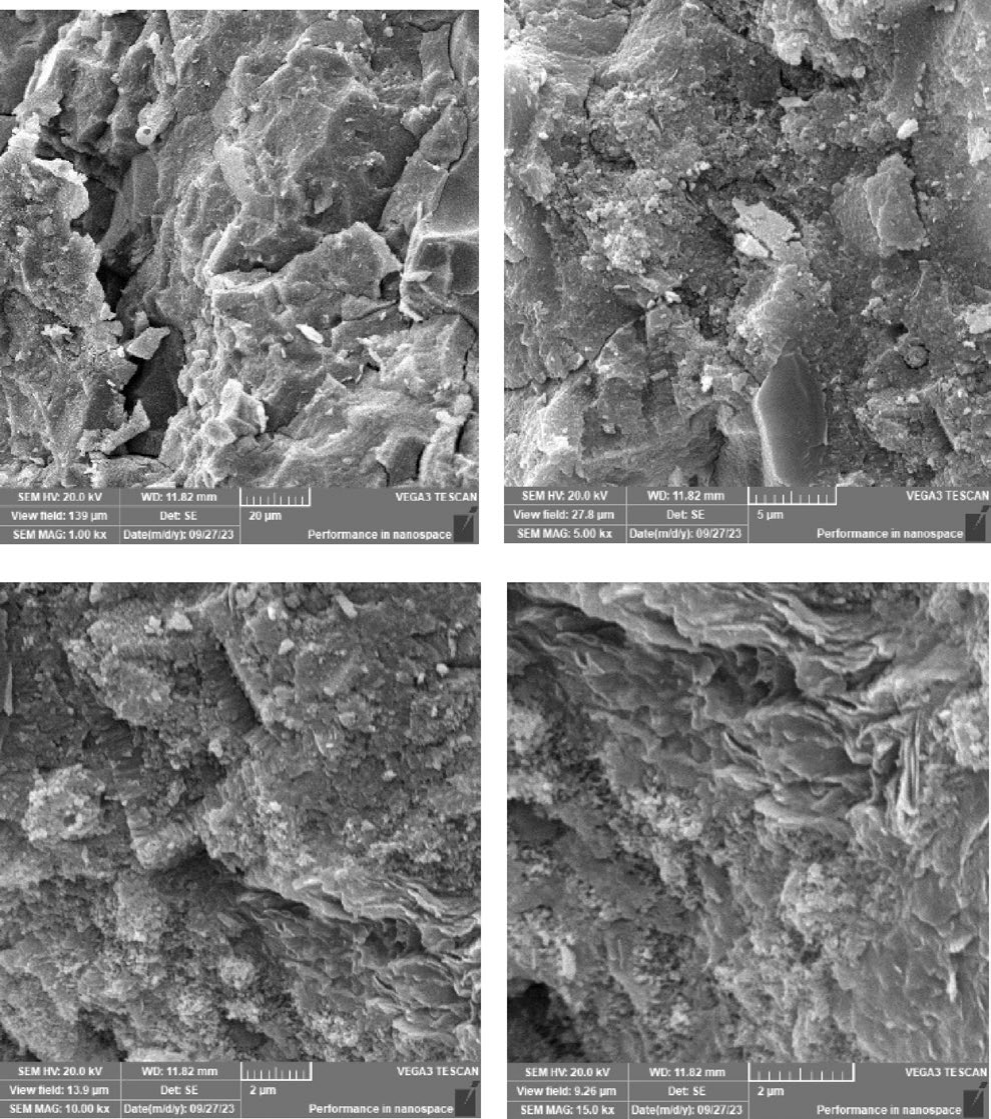
SEM schematic diagram for slag + 5%FA + 10% B + 15% RHA with sodium hydroxide + sodium silicate + sodium carbonate.

**Fig. 15:**
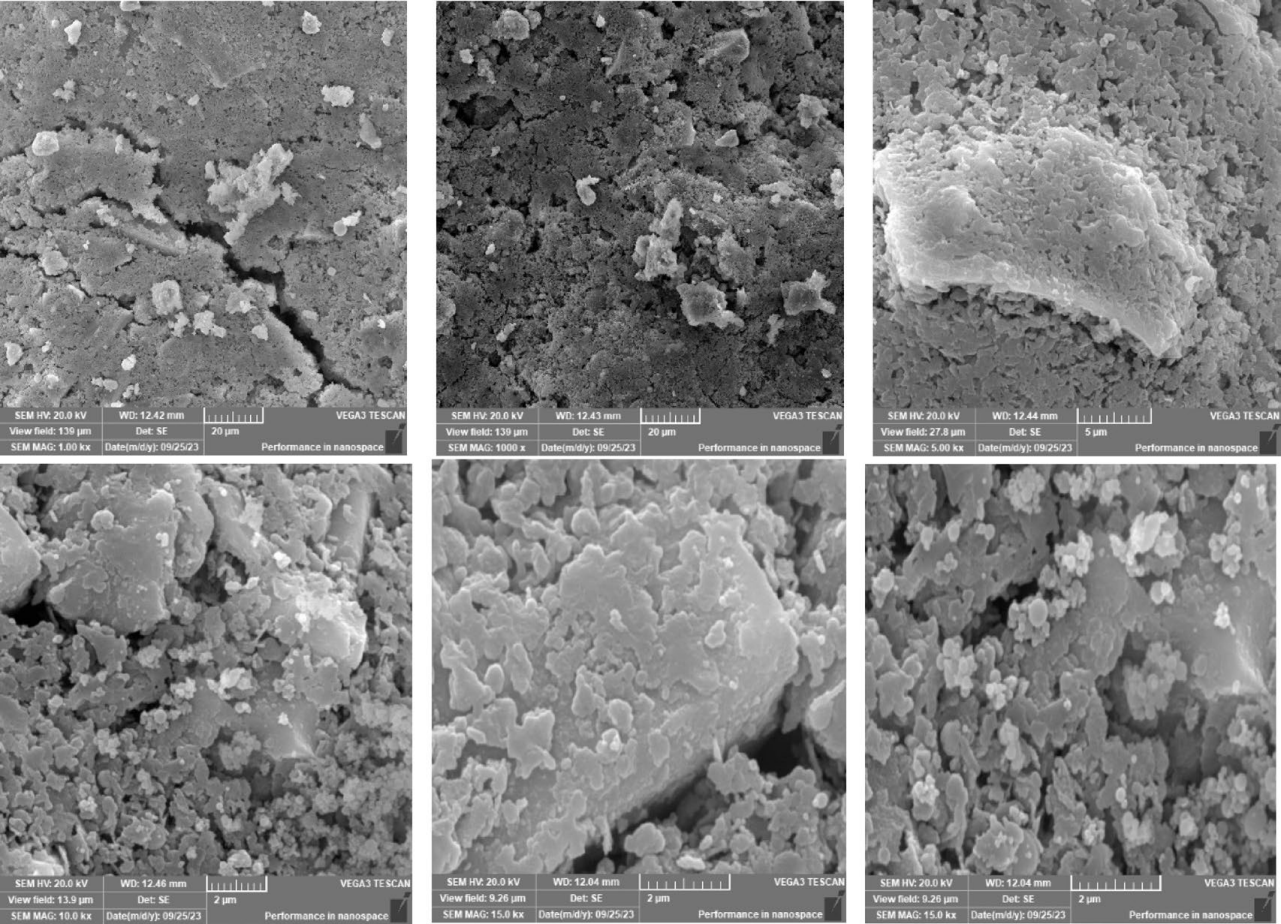
SEM schematic diagram for slag + 5%FA + 10% B + 15% RHA with sodium silicate.

XRD as per previous literature revealed amorphous humps indicative of alkali activated gel. The mechanism involves: (1) Alkali dissolution, (2) Polycondensation, (3) C-A-S-H/N-A-S-H gel formation^36^. C-A-S-H (Ca/Si > 1.0) and N-A-S-H (Al/Si > 0.5) were identified via EDAX ratios^36^.

**Fig. 16:**
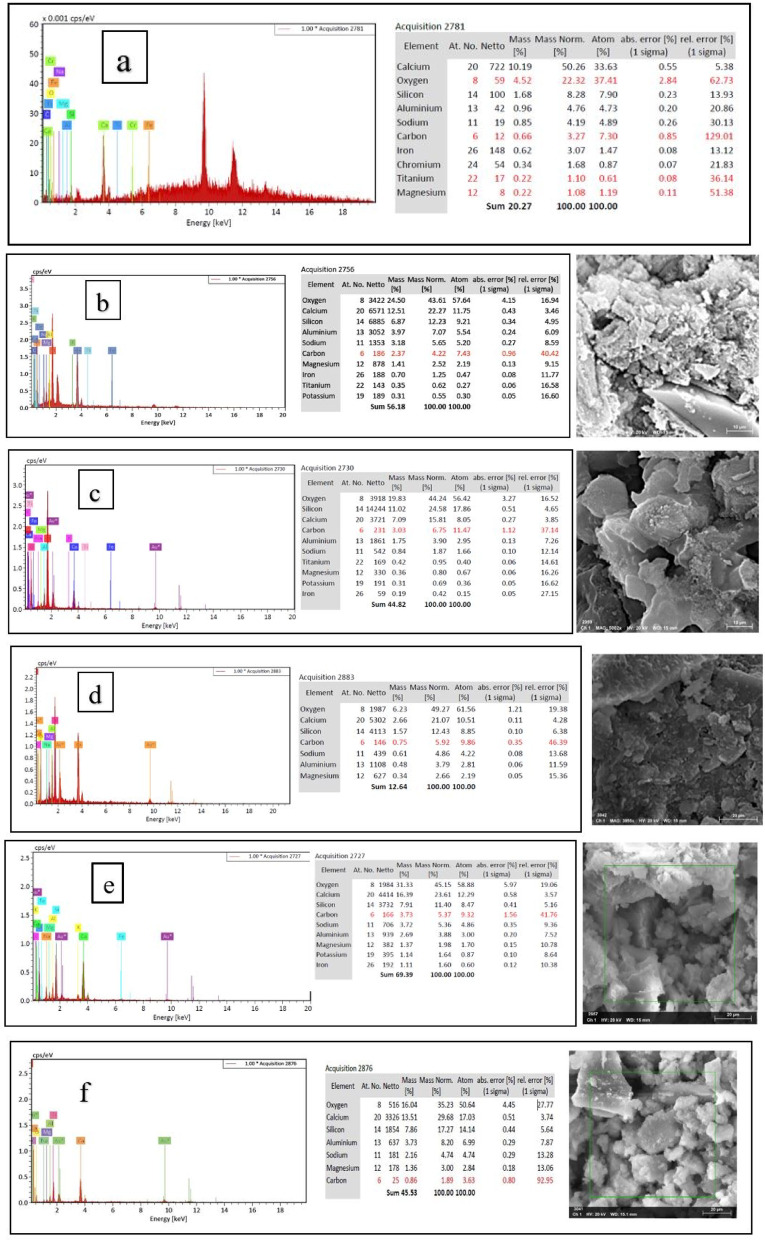

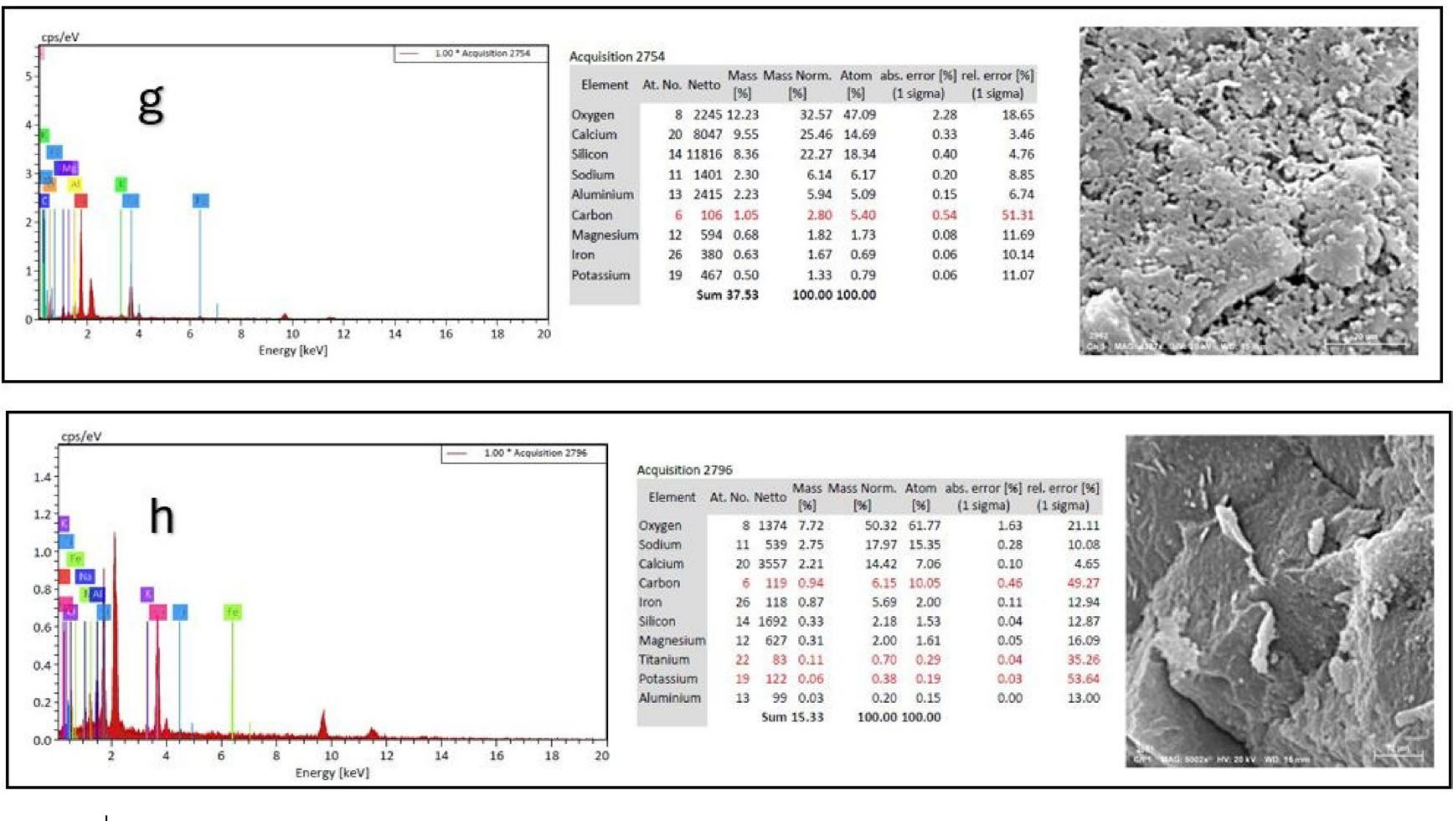
Index images of (**a**) Slag + 10% B with Sodium Silicate; (**b**) Slag + 10% B with Sodium Silicate + Sodium Carbonate; (**c**) Slag + 15% FA with Sodium Silicate; (**d**) Slag + 15% RHA with Sodium Silicate; (**e**) Slag + 15% RHA with Sodium Silicate + Sodium Carbonate; (**f**) Slag + 5% FA + 10% B + 15% RHA with Sodium Hydroxide; (**g**) Slag + 5% FA + 10% B + 15% RHA with Sodium Silicate; (**h**) Slag + 5% FA + 10% B + 15% RHA with Sodium Hydroxide + Sodium Silicate + Sodium Carbonate.

**Fig. 17:**
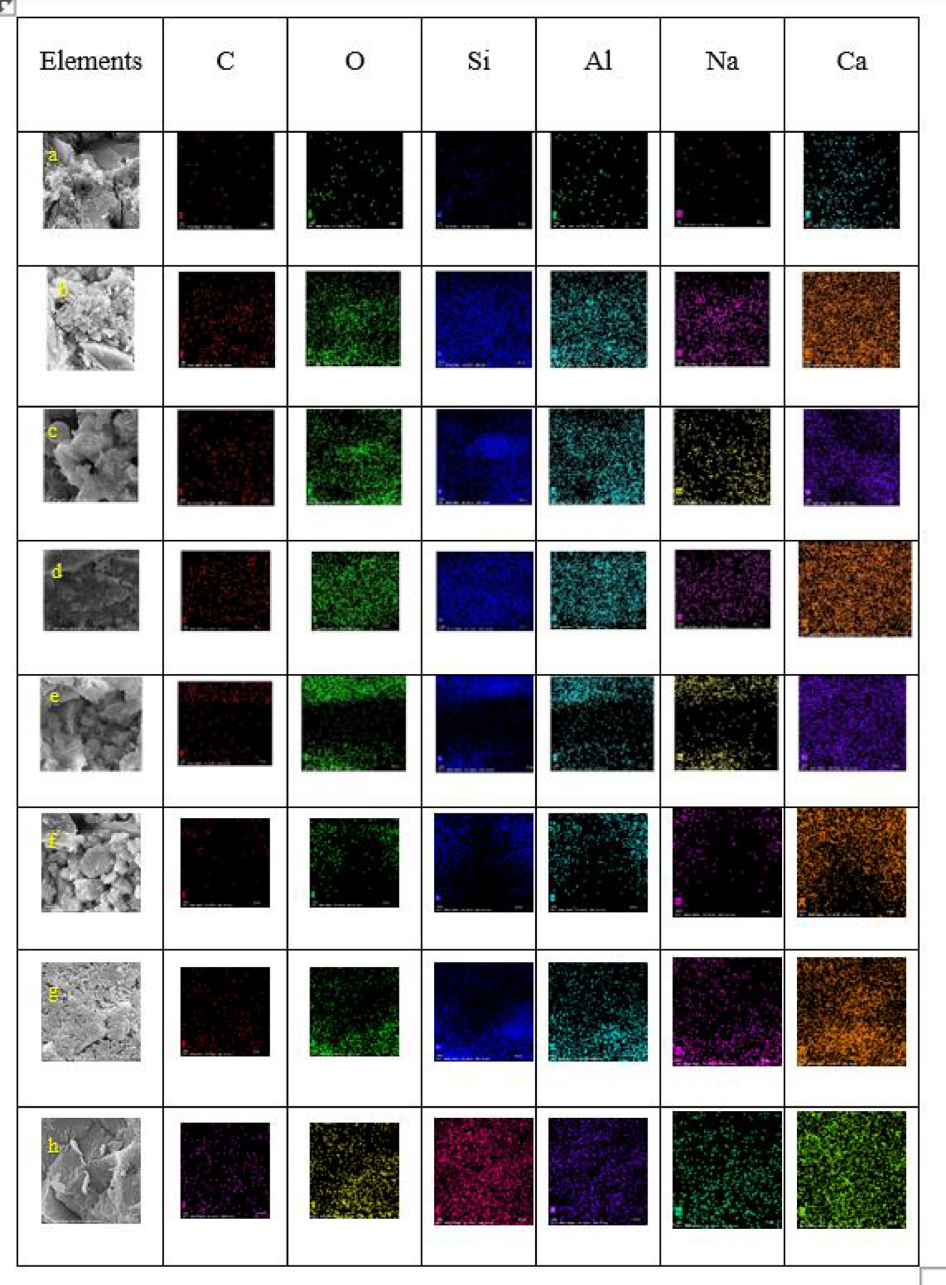
Elemental maps of (**a**) Slag + 10% B with Sodium Silicate; (**b**) Slag + 10% B with Sodium Silicate + Sodium Carbonate; (**c**) Slag + 15% FA with Sodium Silicate; (**d**) Slag + 15% RHA with Sodium Silicate; (**e**) Slag + 15% RHA with Sodium Silicate + Sodium Carbonate; (**f**) Slag + 5% FA + 10% B + 15% RHA with Sodium Hydroxide; (**g**) Slag + 5% FA + 10% B + 15% RHA with Sodium Silicate; (**h**) Slag + 5% FA + 10% B + 15% RHA with Sodium Hydroxide + Sodium Silicate + Sodium Carbonate.

## Conclusions

The analyses conducted in this study yield several key conclusions regarding the performance and characteristics of one-part alkali activated concrete:

### Compressive strength

The limited effectiveness of sodium carbonate as a standalone activator for slag-based alkali activated concrete underscores the critical function of alkaline activators in the alkali activatedization process. The synergistic combination of sodium silicate and sodium carbonate produced a modest but significant increase in compressive strength, highlighting the intricate interactions between these activator components that require further investigation. Variations in compressive strength are attributed to distinct chemical reactions and mechanisms involved in alkali activatedization, emphasizing the pivotal role of alkaline activators on reaction kinetics and product formation.

Recent advances in ternary activators highlight synergies between silica and carbonate phases^38^.

### Optimal replacement ratios

The optimal replacement ratios identified—5% fly ash, 10% bentonite, and 15% rice husk ash by weight of slag—constitute an effective blend of precursor materials that enhances the compressive strength of one-part alkali activated concrete. The consistency of these optimal ratios across different activator formulations demonstrates the robustness of these findings and their potential applicability in diverse construction contexts.

### Activator efficiency

Sodium silicate exhibited superior performance, yielding a 40% increase in 28-day compressive strength compared to sodium hydroxide. This can be attributed to its dual role as a source of reactive silica and alkali ions, which together facilitate alkali activatedization. The maximum compressive strength of 47 N/mm² achieved with a ternary activator blend (sodium silicate, sodium hydroxide, and sodium carbonate in a 6:3:1 ratio) illustrates the potential for optimizing activator combinations to fully leverage the capabilities of one-part alkali activated concrete. Notably, enhancements of 57% and 12% in compressive strength over sodium hydroxide and sodium silicate, respectively, underscore the transformative impact of this optimized activator combination on mechanical performance.

### Microstructural analysis (SEM and EDAX)

Comprehensive microstructural and elemental analyses via Scanning Electron Microscopy (SEM) and Energy-Dispersive X-ray Spectroscopy (EDAX) elucidate the complex relationships between precursor materials, activator selection, and their collective influence on microstructural development and compressive strength. The slag-only alkali activated demonstrated a dense microstructure characterized by C–A–S–H and N–A–S–H gel phases, indicating effective activation by sodium silicate. The inclusion of bentonite resulted in a more heterogeneous microstructure with increased aluminum content, suggesting the formation of a complex N–A–S–H gel structure. In contrast, the slag-fly ash alkali activated exhibited a less dense microstructure dominated by N–A–S–H gel due to pozzolanic reactions.

The combination of slag and rice husk ash led to a porous microstructure with silica-rich phases, indicating a mixed gel structure. The multi-precursor alkali activated revealed a highly heterogeneous microstructure with diverse hydration products, including C–A–S–H and N–A–S–H, reflecting the complex interactions among precursor materials. Notably, the choice of activator significantly influenced hydration reactions, with sodium silicate-activated samples showing a denser microstructure, while sodium carbonate-activated samples were more porous. The combination of sodium silicate and sodium carbonate integrated characteristics of both activators, suggesting a nuanced interplay of hydration products.

In conclusion, the findings from mechanical testing and microstructural analyses provide a comprehensive understanding of the development and composition of one-part alkali activated concrete. This study highlights the significance of precursor materials and activator selection in optimizing hydration reactions and overall performance. The insights gained can guide the design of sustainable, high-performance construction materials, thereby contributing to advancements in building engineering and addressing the evolving demands of the construction industry. Further research referencing published studies will be essential to validate and expand upon these findings, ensuring their relevance and applicability in real-world scenarios.

## Data Availability

The datasets used and/or analyzed during the current study available from the corresponding author on reasonable request.

## References

[1] Miller, S. A., Horvath, A. & Monteiro, P. J. Readily implementable techniques can cut annual CO_2_ emissions from the production of concrete by over 20%. *Environ. Res. Lett.* **11** (7), 074029 (2016).

[2] Shen, W. et al. Cement industry of china: driving force, environment impact and sustainable development. *Renew. Sustain. Energy Rev.* **75**, 618–628 (2017).

[3] Zhang, C. Y., Han, R., Yu, B. & Wei, Y. M. Accounting process-related CO_2_ emissions from global cement production under shared socioeconomic pathways. *J. Clean. Prod.* **184**, 451–465 (2018).

[4] Ali, M. B., Saidur, R. & Hossain, M. S. A review on emission analysis in cement industries. *Renew. Sustain. Energy Rev.* **15** (5), 2252–2261 (2011).

[5] Shekhovtsova, J. et al. Estimation of fly Ash reactivity for use in alkali-activated cements-a step towards sustainable building material and waste utilization. *J. Clean. Prod.* **178**, 22–33 (2018).

[6] Shi, C., Shi, Z., Hu, X., Zhao, R. & Chong, L. A review on alkali-aggregate reactions in alkali-activated mortars/concretes made with alkali-reactive aggregates. *Mater. Struct.* **48**, 621–628 (2015).

[7] Vishwakarma, V. & Ramachandran, D. Green concrete mix using solid waste and nanoparticles as alternatives—A review. *Constr. Build. Mater.* **162**, 96–103 (2018).

[8] Nematollahi, B., Sanjayan, J. & Shaikh, F. U. A. Synthesis of heat and ambient cured one-part alkali activated mixes with different grades of sodium silicate. *Ceram. Int.* **41** (4), 5696–5704 (2015).

[9] Assi, L., Carter, K., Deaver, E. E., Anay, R. & Ziehl, P. Sustainable concrete: Building a greener future. *J. Clean. Prod.* **198**, 1641–1651 (2018).

[10] van Deventer, J. S., Provis, J. L., Duxson, P. & Brice, D. G. Chemical research and climate change as drivers in the commercial adoption of alkali activated materials. *Waste Biomass Valoriz.* **1**, 145–155 (2010).

[11] Humbert, P. S. & Castro-Gomes, J. CO2 activated steel slag-based materials: A review. *J. Clean. Prod.* **208**, 448–457 (2019).

[12] Zhang, L. Production of bricks from waste materials—A review. *Constr. Build. Mater.* **47**, 643–655 (2013).

[13] Sandanayake, M., Gunasekara, C., Law, D., Zhang, G. & Setunge, S. Greenhouse gas emissions of different fly Ash based alkali activated concretes in Building construction. *J. Clean. Prod.* **204**, 399–408 (2018).

[14] Qin, L., Gao, X. & Chen, T. Recycling of raw rice husk to manufacture magnesium oxysulfate cement based lightweight building materials. *J. Clean. Prod.* **191**, 220–232 (2018).

[15] Tan, H. et al. Utilization of lithium slag by wet-grinding process to improve the early strength of sulphoaluminate cement paste. *J. Clean. Prod.* **205**, 536–551 (2018).

[16] Bernal, S. A. & Provis, J. L. Durability of alkali-activated materials: progress and perspectives. *J. Am. Ceram. Soc.* **97** (4), 997–1008 (2014).

[17] Hojati, M. & Radlińska, A. Shrinkage and strength development of alkali-activated fly ash-slag binary cements. *Constr. Build. Mater.* **150**, 808–816 (2017).

[18] Yazdi, M. A., Liebscher, M., Hempel, S., Yang, J. & Mechtcherine, V. Correlation of microstructural and mechanical properties of alkali activateds produced from fly Ash and slag at room temperature. *Constr. Build. Mater.* **191**, 330–341 (2018).

[19] Provis, J. L. Activating solution chemistry for alkali activateds. In *Alkali Activateds*, 50–71. (Woodhead Publishing, 2009).

[20] Somna, K., Jaturapitakkul, C., Kajitvichyanukul, P. & Chindaprasirt, P. NaOH-activated ground fly Ash alkali activated cured at ambient temperature. *Fuel*. **90** (6), 2118–2124 (2011).

[21] Oderji, S. Y., Chen, B. & Jaffar, S. T. A. Effects of relative humidity on the properties of fly ash-based alkali activateds. *Constr. Build. Mater.* **153**, 268–273 (2017).

[22] Cheah, C. B., Samsudin, M. H., Ramli, M., Part, W. K. & Tan, L. E. The use of high calcium wood ash in the preparation of ground granulated blast furnace slag and pulverized fly ash alkali activateds: A complete microstructural and mechanical characterization. *J. Clean. Prod.* **156**, 114–123 (2017).

[23] Mehta, A. & Siddique, R. Sustainable alkali activated concrete using ground granulated blast furnace slag and rice husk ash: strength and permeability properties. *J. Clean. Prod.* **205**, 49–57 (2018).

[24] Zhuang, X. Y. et al. Fly ash-based alkali activated: clean production, properties and applications. *J. Clean. Prod.* **125**, 253–267 (2016).

[25] Duxson, P. & Provis, J. L. Designing precursors for alkali activated cements. *J. Am. Ceram. Soc.* **91** (12), 3864–3869 (2008).

[26] Adesanya, E., Ohenoja, K., Luukkonen, T., Kinnunen, P. & Illikainen, M. One-part alkali activated cement from slag and pretreated paper sludge. *J. Clean. Prod.* **185**, 168–175 (2018).

[27] Luukkonen, T., Abdollahnejad, Z., Yliniemi, J., Kinnunen, P. & Illikainen, M. One-part alkali-activated materials: A review. *Cem. Concr. Res.* **103**, 21–34 (2018).

[28] Rashad, A. M. A comprehensive overview about the influence of different admixtures and additives on the properties of alkali-activated fly ash. *Mater. Des*. **53**, 1005–1025 (2014).

[29] Saha, S. & Rajasekaran, C. Enhancement of the properties of fly Ash based alkali activated paste by incorporating ground granulated blast furnace slag. *Constr. Build. Mater.* **146**, 615–620 (2017).

[30] Shang, J. et al. Alternation of traditional cement mortars using fly ash-based alkali activated mortars modified by slag. *J. Clean. Prod.* **203**, 746–756 (2018).

[31] Lee, N. K. & Lee, H. K. Setting and mechanical properties of alkali-activated fly ash/slag concrete manufactured at room temperature. *Constr. Build. Mater.* **47**, 1201–1209 (2013).

[32] Temuujin, J. V., Van Riessen, A. & Williams, R. Influence of calcium compounds on the mechanical properties of fly Ash alkali activated pastes. *J. Hazard. Mater.* **167** (1–3), 82–88 (2009). 19201089 10.1016/j.jhazmat.2008.12.121

[33] Perná, I. & Hanzlíček, T. The setting time of a clay-slag alkali activated matrix: the influence of blast-furnace-slag addition and the mixing method. *J. Clean. Prod.* **112**, 1150–1155 (2016).

[34] Ma, C., Zhao, B., Guo, S., Long, G. & Xie, Y. Properties and characterization of green one-part alkali activated activated by composite activators. *J. Clean. Prod.* **220**, 188–199 (2019).

[35] Oderji, S. Y., Chen, B., Ahmad, M. R. & Shah, S. F. A. Fresh and hardened properties of one-part fly ash-based alkali activated binders cured at room temperature: effect of slag and alkali activators. *J. Clean. Prod.* **225**, 1–10 (2019).

[36] Luukkonen, T., Abdollahnejad, Z., Yliniemi, J., Kinnunen, P. & Illikainen, M. Comparison of alkali and silica sources in one-part alkali-activated blast furnace slag mortar. *J. Clean. Prod.* **187**, 171–179 (2018).

[37] Azevedo, A. G. S. & Strecker, K. Kaolin, fly-ash and ceramic waste based alkali-activated materials production by the one-part method. *Constr. Build. Mater.* **269**, 121306 (2021).

[38] Samadhiya, A., Bhunia, D., Chakraborty, S. & Lahoti, M. *Influence of Activator Ratios and Concentration on the Physio-mechanical and Microstructural Characteristics of the Alkali Activateds Derived from Sandstone Processing Waste*, 1–16 (Environmental Science and Pollution Research, 2024). 10.1007/s11356-024-33019-0 38526714

[39] Samadhiya, A., Bhunia, D. & Chakraborty, S. Alkali-activation potential of sandstone wastes with electric arc furnace slag as co-additive. *Arab. J. Sci. Eng.* **49** (4), 5817–5833 (2024).

[40] Samadhiya, A., Bhunia, D., Chakraborty, S. & Lahoti, M. *Optimization of Sandstone Processing Waste, Electric Arc Furnace Slag, and Fly Ash-based Ternary Blended Eco-friendly Alkali Activateds*, 1–22 (Environmental Science and Pollution Research, 2024). 10.1007/s11356-024-35610-x 39585565

[41] Samadhiya, A., Bhunia, D., Chakraborty, S. & Kaushik, T. Alkali-activation potential of stone wastes. *Mater. Today Proc*. (2023).

